# Glucocorticoid use and perceptions of side effects among patients with rheumatic medical diseases: Insights from a developing country

**DOI:** 10.1371/journal.pone.0327436

**Published:** 2025-07-03

**Authors:** Fatima Alnaimat, Hamza Alduraidi, Ali Rezeq Ali Yaghi, Mohammad Mahmoud Tarbiah, Asim Khanfar, Moath Abusheikha, Ali M. Hamad

**Affiliations:** 1 Department of Internal Medicine, Division of Rheumatology/ School of Medicine -University of Jordan, Amman, Jordan; 2 School of Nursing, The University of Jordan, Amman, Jordan; 3 School of Medicine, The University of Jordan, Amman, Jordan; 4 Department of Surgery, King Hussein Cancer Center, Amman, Jordan; 5 Department of Internal Medicine, Rochester General Hospital, Rochester, New York, United States of America; 6 Surgery Department, Airedale NHS Foundation Trust, West Yorkshire, United Kingdom; 7 Trauma and Orthopaedics Department - The Royal London Hospital, London, United Kingdom; Shahid Beheshti University of Medical Sciences School of Medicine, ISLAMIC REPUBLIC OF IRAN

## Abstract

Chronic glucocorticoid (GC) therapy is common in patients with rheumatic medical diseases (RMD). However, long-term use of GCs can be associated with significant adverse effects. This study aims to determine the prevalence of GC use among patients with RMD and understand their perceptions of GC use and safety. This cross-sectional survey was conducted at a tertiary academic university hospital in Amman, Jordan. RMD patients were anonymously approached while awaiting rheumatology clinic appointments. Convenience sampling was employed, with enrollment taking place between January and September 2021. Of 500 participants, 315 (63%) reported current (171/315, 54.3%) or past (144/315, 45.7%) use of GCs, primarily prescribed for RMD (267/315, 84.7%). Most (270/315, 85.7%) used GCs orally, with the majority taking them daily (266/315, 84.4%). A small percentage (33/315, 10.5%) used GCs only as needed. Long-term use was common, with 57.8% (182/315) reporting use for years and 84.1% (265/315) adhering to doctor recommendations. Perception-wise, 69.5% (219/315) of GC users believed in its efficacy, whereas 78.7% (248/315) considered it unsafe, compared to 51.9% (96/185) of non-users. Awareness of side effects was higher among GC users (219/315, 69.5%) than non-users (79/185, 42.7%), with weight gain being the most reported side effect. Media and personal research were the primary information sources for GC users (193/315, 61.3%). Side effects were reported by 110/315 (34.9%) GC users, with weight gain as the most common (47%). Both perceived efficacy and safety of GC were positively and significantly correlated with educational level (p = .005, p = .000, respectively) and monthly income (p = .044, p = .000, respectively). GC use is prevalent among Jordanian patients with RMDs, with perceptions of efficacy and safety strongly influenced by education and income. Enhanced patient education on the side effects of GC is crucial for improving treatment adherence and outcomes.

## Introduction

Since their discovery in the mid-nineteenth century, glucocorticoids (GCs) have been an integral component of therapy for many diseases across several medical fields, particularly for autoimmune diseases due to their immunosuppressive and immunomodulatory effects [1,2]. Short-term GCs are often deemed necessary to alleviate symptoms in inflammatory arthritis, such as rheumatoid arthritis (RA), before the onset of action of disease-modifying antirheumatic drugs (DMARDs) [3]. In other rheumatic medical diseases (RMDs), such as systemic lupus erythematosus, vasculitis, polymyalgia rheumatic, and inflammatory myositis, GCs continue to be a key component of treatment [4,5].

GCs have a plethora of side effects that range from mild to more serious, such as hyperglycemia, osteoporosis, fractures, and infection, among others [6–8]. When administered properly and for a limited time, the benefits of GCs predominate with few negative effects, while inappropriate dosing, prolonged usage, or abrupt withdrawal might cause major adverse outcomes. Consequently, current practice and international society guidelines on the use of GC recommend that their use should be at the lowest possible dose for the shortest possible duration to prevent such adverse events [9,10].

The high costs of DMARDs and biologic drugs, which are considered a standard of care for many RMDs, may limit their use in some patients, particularly in developing countries [11]. Compared to neighboring countries in the region with higher national gross domestic product (GDP) per capita, such as Saudi Arabia and the United Arab Emirates, Jordanian patients with RA were found to have a significantly higher usage of GCs and substantially lower use of tumor necrosis factor (TNF) inhibitors and methotrexate [12].

Multiple factors can influence the onset of side effects of GCs, most importantly the dose and duration of administration, which has led to conflicting evidence regarding safe dosages [13]. Significant side effects from glucocorticoid (GC) therapy can impact patient adherence in conditions where GCs are crucial. Conversely, some patients may perceive GCs as a safer option compared to more aggressive treatments like biologics and may continue using them despite medical advice to the contrary. This issue arises from differing perceptions of side effects between patients and prescribers [14].

This study aims to determine the prevalence and pattern of GC use in a sample of Jordanian patients with RMD, identify their perceptions of safety and efficacy regarding GC use and its associated side effects, and evaluate correlations between awareness of GC therapy and various sociodemographic factors.

## Methods

### Study design

This cross-sectional survey was conducted at the outpatient rheumatology clinic of Jordan University Hospital (JUH), a tertiary care center in Amman, Jordan, which serves a large portion of the Jordanian population. In 2020, the hospital recorded over 400,000 outpatient visits [15]. The study was conducted between January 1, 2021, and September 30, 2021, with a brief interruption due to COVID-19 lockdown measures. A trained research assistant identified and approached eligible patients while awaiting their appointments. Interviews were conducted during weekday morning clinic sessions without the presence of the patient’s treating physician to minimize potential bias.

A structured questionnaire was developed by designing and testing on a sample of patients and modified as needed. The final questionnaire comprised four sections: (1) sociodemographic data; (2) patient-specific characteristics, including diagnosis and current medications; (3) glucocorticoid (GC) usage patterns, including self-reported adherence; and (4) awareness and presence of GC-related side effects. The study followed guidance on survey design and reporting from Zimba and Gasparyan [16], and we adhered to the STROBE guidelines for reporting cross-sectional studies [17].

### Population and sample

The sampling frame of the study included all patients of JUH’s rheumatology outpatient clinics during the period above. The sample size was calculated using Cochran’s Sampling Techniques for cross-sectional surveys with proportional outcomes (Kotrlik), where the type 1 error margin, alpha, was set at.05, the expected proportion of the population, p, was set at.50, and absolute error or precision, d, was set at.05. The calculation yielded a minimum required sample size of 500 subjects. Convenience sampling was employed, and patients were included sequentially as they accepted to enroll in the study. Inclusion criteria included any patient who presented to the rheumatology clinic for a first or follow-up visit and was willing to participate by giving verbal consent and agreeing to answer the research questions. If patients were younger than 18 years, consent was obtained from the patient’s family members accompanying the patient.

### Ethics and approval

This study was conducted in accordance with the Declaration of Helsinki and approved by the IRB of Jordan University Hospital (IRB: 10/2020/8621). Before enrolling in the study, patients received a brief overview of its aims and were informed of the survey’s anonymity and their right to withdraw from it at any moment. Verbal consent was obtained from the participants and was witnessed by the research assistants.

### Statistical analysis

Data entry, preparation, and statistical analysis were conducted using SPSS (Statistical Package for Social Sciences) version 23. Faulty responses were omitted, and errors during data entry were avoided by frequently re-checking the original data. We used descriptive statistics such as mean, median, and standard deviation to describe continuous quantitative variables. For categorical variables, we used frequencies and percentages. Bivariate correlations between categorical/ordinal variables were tested for magnitude, direction, and statistical significance using Spearman’s correlation coefficient, where a p-value of <.05 was considered statistically significant.

## Results

### Sample characteristics

Out of 500 study participants, 443 were female (88.6%), and 57 were male (11.4%) (Table 1). Patients younger than 50 comprised 46.4% of the sample (n = 232), while 53.6% were above 50 (n = 268). Most patients lived in urban areas (350/500, 76%), and most presented for follow-up visits (388/500, 77.6%), while the remaining patients were first-time visitors (112/500, 22.4%).

**Table 1:** Sociodemographics of the study sample (n = 500).

Gender	Number and Percentage
Male	57 (11.40%)
Female	443 (88.60%)
Age	
<16	22 (4.40%)
16-25	26 (5.2%)
26-40	80 (16%)
41-50	102 (20.40%)
51-60	149 (29.80%)
>60	119 (23.80%)
Marital Status	
Single	91 (18.20%)
Married	356 (71.20%)
Divorced	5 (1%)
Widow	48 (9.60%)
Occupation	
Working	96 (19.2%)
Not working	404 (80.80%)
Residential Area	
Urban	380 (76.00%)
Rural	120 (24.00%)
Educational level	
No formal education	15 (3%)
ISCED 1	137 (27.40%)
ISCED 3	115 (23%)
ISCED 4 or 5	111 (22.20%)
ISCED 6	103 (20.60%)
ISCED 7	15 (3.00%)
ISCED 8	4 (0.80%)
Monthly income	
<400 JOD**	144 (28.80%)
400-1000 JOD	336 (67.20%)
>1000 JOD	20 (4.00%)
Diagnosis	
Inflammatory arthritis	138 (27.60%)
Connective tissue disease	55 (11.00%)
Osteoarthritis	34 (6.80%)
Chronic bone pain	29 (5.80%)
Fibromyalgia	24 (4.80%)
Osteoporosis	19 (3.80%)
Vasculitis	11 (2.20%)
Gout	8 (1.60%)
Multiple diagnoses	59 (11.80%)
Others	123 (24.6)

Abbreviations: * International Standard Classification of Education, ** Jordanian dinar.

Based on the International Standard Classification of Education (ISCED) [18], the educational levels of the participants were as follows: 3% had no formal education, 27.4% completed ISCED 1 (Primary Education), 23% completed ISCED 3 (Upper Secondary Education), 22.2% completed ISCED 4 or 5 (Post-secondary non-Tertiary or Short-cycle Tertiary Education), 20.6% held ISCED 6 (Bachelor’s degree), 3% held ISCED 7 (Master’s degree), and 0.8% held ISCED 8 (Doctoral degree). When asked about the monthly income, 336 out of 500 patients (67.2%) reported having a household income that falls in the middle-income range of JD 400–1,000 per month (1 JD = 1.40 USD).

RMDs varied in the study sample; 387 (77.4%) had a musculoskeletal diagnosis including inflammatory arthritis in 138 patients (27.6%), connective tissue disease in 55 patients (11%), osteoarthritis in 34 patients (6.8%), chronic bone pain in 29 patients (5.8%), fibromyalgia in 24 patients (4.8%) and osteoporosis in 19 patients (3.8%).

### Glucocorticoid use among the study sample

Of 500 participants, 315 declared current (171/315, 54.3%) or past use of GCs (144/315, 45.7%). Among these 315 participants, 267 (84.7%) were prescribed GCs to treat their RMDs, whereas 48/315 (15.2%) took the drug for non-rheumatological diseases. The most common route of GC administration among GC users was the oral route in 270/315 (85.7%), which was mostly a daily use in 266/315, 84.4%. Interestingly, 10.5% of the patients (33 out of 315) reported using GCs only as needed, based on their perception of their condition. More than half of the GC users reported taking the drug for years (182/315, 57.8%), whereas 48/315 (15.2%) of the patients reported a duration of less than one month, 63/315 (20%) for 1–3 months, and 20/315 (6.3%) for more than three months. Among patients with current or past glucocorticoid (GC) use, 84.1% (265/315) stated they always follow their doctor’s recommendations for starting or stopping GC treatment. Meanwhile, 9.2% (29/315) reported often following medical advice, 5.7% (18/315) sometimes do, and 0.9% (3/315) rarely adhere to medical guidance, choosing instead to follow their judgment.

### Patient perception of the efficacy, safety, and awareness of potential GC side effects

#### Efficacy of GC.

The entire 500 patients sample was asked for their opinions on the efficacy of GC therapy, with comparisons made between GC users and non-users (Table 2). Among GC users, 69.5% (219/315) think it is efficacious, whereas 11.4% (36/315) do not think it’s efficacious, and 19% (60/315) were unsure. In contrast, among non-users, 34.7% (64/185) think it is efficacious, whereas 7.7% (14/185) disagree, and 57.8% (107/185) are unsure.

**Table 2:** Perceptions of the efficacy and safety of glucocorticoids among the study participants.

	GC*-users (n = 315) Number and percentage	Non-users (n = 185) Number and percentage	P value
Type of visit			
First Visit	44(14%)	68(36.8%)	
Follow up	271(86%)	117(63.2%)	
Is GC therapy safe			
Yes	22 (7%)	6(3.2%)	
No	248(78.7%)	96(51.9%)	
Unsure	45 (14.3%)	83(44.7%)	
Is GC therapy Efficacious?			
Yes	219(69.5%)	64(34.7)	
No	36 (11.4%)	14(7.7)	
Unsure	60(19.0%)	107(57.8%)	
Are you aware of GC’s adverse effects?			
Yes	216(68.6%)	79(42.7%)	
No	89(28.3%)	95(51.4%)	
Not sure	10(3.2%)	11(5.9%)	
Are you aware that GCs may cause the following adverse effects?			
Weight gain	156 (49.5%)	72 (39%)	
Osteoporosis	118 (37.5%)	32 (17.3%)	
LL edema**	52 (16.5%)	23(12.4%)	
Hyperglycemia	44 (14%)	6 (3.2%)	
High blood pressure	30 (9.5%)	5 (2.7%)	
Risk of infections	26(8.3%)	10 (5.4%)	
Myopathy	25(7.9%)	3(1.6%)	
Cataract	16(5.1%)	4(2.2%)	
Mood disorders	15(4.8%)	2(1.1%)	
Sleep disturbances	14(4.4%)	2(1.1%)	
Skin thinning	13(4.1%)	7(3.8%)	
Source of information regarding GC adverse effects			
Physician	19(6%)	2(1.1%)	
Pharmacist	2(0.6%)	0(0%)	
Family member	17(5.4%)	32(17.3%)	
Media/personal reading	193(61.3%)	57(30.1%)	

*GC: Glucocorticoid.

** LL: lower limb.

#### Safety of GC.

Interestingly, most GC users think that GC therapy is unsafe (248/315, 78.7%), compared to 51.9% of non-users (96/185). The percentage of participants who were unsure whether GC therapy was safe or not was 14.3% for GC users (45/315) and 44.7% (83/185) for non-users (Table 2).

#### Awareness of potential GC side effects.

More than half of GC users (219/500, 69.5%) reported that they were aware that GC therapy causes several side effects, in contrast to 79/185(42.7%) of those with no prior use of GC. Most non-users of GC reported they do not know if GC causes adverse effects (95/185, 51.4%). Among several GC side effects the patient sample was asked about, weight gain was the most popular, chosen by 49.5% of GC users and 39% of non-GC users. Table 2 shows the participants’ views on several other potential adverse effects of GC.

Regarding sources of information about GC adverse effects, the vast majority of patients who use GC reported it came from the media or their reading (193/315,61.3%), which was also the major source in 30.1% of non-GC therapy users. Unfortunately, physicians and pharmacists were the data sources for a negligible percentage of the study participants.

#### Real side effects experienced following GC use.

**Fig 1:**
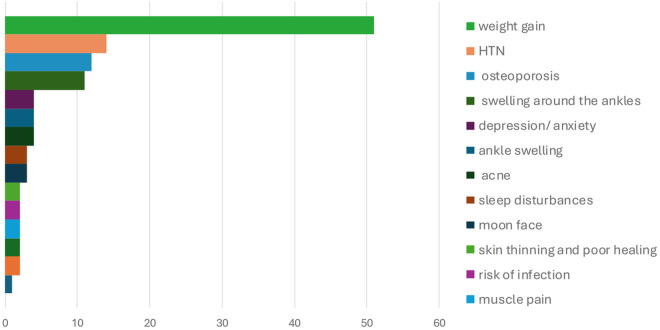
The prevalence of the real-time adverse effects encountered by the GC users (n = 315).

When asked if any adverse effects were experienced after GC use, 110/315 participants (34.9%) reported suffering from side effects. Fig 1 summarizes the main side effects experienced by participants. The most reported side effect was weight gain among 51 participants (47%). Other less commonly reported side effects included depression, sleep disturbance, cataracts, hyperlipidemia, poor wound healing, thinning of skin, acne, moon face, and muscle pain.

**Correlation between socio-demographic characteristics and perceived efficacy and safety of GC** A series of Spearman’s bivariate correlation tests were conducted to examine correlations between the perceived efficacy and safety of GCs and the socio-demographic characteristics of gender, age, marital status, occupation, place of residence, educational level, and monthly income (Table 3). Both perceived efficacy and safety of GC were positively and significantly correlated with educational level (p = .005,.000, respectively) and monthly income (p = .044,.000, respectively). At the same time, none of the other characteristics was found to be significantly correlated with the perceived efficacy or safety of GCs.

**Table 3:** Correlation between Socio-demographics and perceived efficacy and safety of GCs.

Characteristic	Perceived Efficacy		Perceived Safety	
Correlation coefficient†	*p*	Correlation coefficient†	*p*
Gender	−.084	.060	.052	.247
Age	.057	.203	−.046	.300
Marital status	−.007	.876	.070	.115
Occupation	−.077	.084	.042	.353
Place of residence	−.009	.834	−.012	.790
Educational level	.126	.005*	.181	.000*
Monthly income	.103	.044*	.156	.000*

† Spearman’s bivariate correlation.

* Statistically significant; p < .05.

## Discussion

In this study, we describe the prevalence of GC use and the perceived safety and efficacy of GC users regarding the adverse effects of GCs in a sample of rheumatology outpatients in Jordan. Our sample found that almost two-thirds of patients reported ever using GCs. Most of those patients had taken oral GCs for rheumatologic diseases. More than half of the GC users reported taking the drug for years.

Slightly more than half of our sample were aware of side effects associated with GCs, with the most popular side effects being weight gain and osteoporosis, and approximately one-third of those who reported ever using GCs exhibited side effects. Awareness about GC adverse effects showed a similar trend in prior studies in the region, with around half of patients from the United Arab Emirates (UAE) who used GCs being aware of side effects such as hypertension, hyperglycemia, and osteoporosis [19]. In a study from Saudi Arabia, the general population also reported similar, albeit lower, rates of awareness of side effects, with almost half of patients reporting weight gain and a third reporting skin changes [20].

Almost one-third of our patients reported exhibiting side effects from their GC therapy, with weight gain being the most commonly reported side effect, followed by osteoporosis and lower extremity swelling. Our findings are similar to those in Saudi Arabia, with the most frequently reported side effect being weight gain, followed by mood changes [20].

The literature indicates that the side effects from GCs typically occur with long-term systemic use, with the most commonly prevalent side effects including hypertension and fractures, according to a systematic review [2]. In contrast, short-term systemic GCs are relatively safer, with some reports of avascular necrosis and severe mood changes [21]. In our sample, more than half of GC users reported use for years. Although we did not directly analyze the correlation between GC duration and perceived safety or efficacy, the high prevalence of long-term GC use in our sample puts patients at risk of side effects. Given the risk of long-term side effects, which are known to increase with the duration and dosage of GCs, international guidelines in rheumatology literature, such as by the European Alliance of Associations for Rheumatology (EULAR), have recommended for rheumatoid arthritis (RA) that GCs be stopped as early as feasible [9]. Therefore, more efforts must be made to educate patients on the tapering of GCs.

While slightly more than half of patients were aware of these side effects, more efforts should be made to improve patient awareness of side effects to ensure compliance with medications, proper follow-up, and early identification. Almost one-third of our sample reported that they had never been informed of side effects by any means. Most of our sample reported knowing about side effects from media or personal reading, and less than twenty percent reported being informed of side effects by healthcare professionals (physicians and pharmacists). In contrast, almost forty percent of the general population in Saudi Arabia were made aware of side effects from healthcare professionals [20]. Despite the vast majority of the patients admitting that they always follow their doctor’s recommendations for starting or stopping GC treatment, there appears to be a gap in patient education in our patient sample, and the lack of communication about side effects from healthcare professionals has the potential to compromise proper patient care.

While the occurrence of potentially harmful side effects is essential when considering prescribing GCs, an arguably equally important factor is understanding what side effects matter most to patients. Studies have shown discrepancies between what side effects are considered most bothersome by patients and physicians. In one study, physicians considered weight gain to be the most bothersome symptom. However, more patients were more likely to report neuropsychiatric symptoms as their most bothersome symptom, whereas physicians significantly underestimated the importance of neuropsychiatric symptoms compared to other side effects [14]. Another study showed that the most important side effects for patients on GCs were weight gain, insomnia, and fatigue, and more clinically significant side effects such as diabetes, cardiovascular disease, and infections were considered less important [22]. A systematic review of patient-reported outcomes in GC use showed that the most important themes for these patients were weight gain, sleep problems, and irritability [23]. In our study, patients were more likely to know that weight gain, osteoporosis, moon face, and swelling are side effects of GC use. Although this question was not a direct indicator of important side effects to patients, better knowledge of these side effects may be a result of fearing these side effects most. Understanding which side effects matter most to patients will help physicians and patients with shared-decision making, which can lead to increased adherence to treatment [24].

In our study, the vast majority of the patients adhered to their physician’s recommendations regarding starting or stopping GC therapy, which is higher than in other studies [14]. There is wide variability in GC compliance across different populations, with some studies ranging around 30–50% [14,25] and others up to 78% adherence [26]. Poor adherence to GCs may stem from the development of side effects, as well as fear about the safety of GCs [25,26]. The safety of GCs remains a controversial topic among physicians and patients, creating a stigma that interferes with disease control; patients who believe GCs are harmful are less likely to be adherent [25]. It’s interesting to note that even though almost three-quarters of our population believed GCs are unsafe, adherence still remains high, which contrasts the aforementioned studies.

Furthermore, it’s interesting to note that our population was adherent despite many patients reporting not receiving information about GC safety from their physicians or pharmacists, considering that healthcare workers would provide positive recommendations. One possible explanation for this increased compliance could be the financial constraints of other therapies, such as DMARDs and biologics in this developing country, which might lead patients to continue GC therapy despite perceptions of unsafety. Cultural differences may also play a role. Further studies are needed to identify barriers to compliance across different populations.

In our study, almost three-quarters of patients believe GCs are unsafe, and less than ten percent consider them safe. These findings were similar to another study in the region, where 62% of UAE patients believed systemic GCs were unsafe [19]. One explanation might be the high percentage of patients who receive information about GCs from the media. One study showed that the influence of the internet and recommendations made by friends and family resulted in misinformation regarding the safety of topical GCs [27]. When patients received their information from physicians, their perceived benefits of GCs significantly increased, whereas those who received information from family, friends, or the media had significantly higher concerns [25].

Even with the perceptions surrounding the safety of GCs, more than half of the patients reported that GCs are efficacious. In our study, the perceived efficacy and safety of GCs correlate significantly with education level and monthly income, which may indicate that patients of higher socioeconomic status were less likely to trust sources that are typically known to spread misinformation. GC safety remains a large focus for physicians, which has led to the recent introduction of the glucocorticoid toxicity index (GTI), a tool used to measure changes in GC toxicity across two points in time [28].

The use of GCs in the long-term treatment of certain inflammatory conditions, such as RA, remains controversial. Although the EULAR has recommended minimizing the use of GCs [9], some studies suggest adding low-dose GCs can remain relatively safe for patients and can provide long-term benefits. In the Glucocorticoid LOw-dose in RheumatoId Arthritis (GLORIA) trial, daily low-dose prednisolone significantly decreased disease activity and limited disease progression in elderly patients over almost two years [29]. Roubille et al. demonstrated that very low-dose GCs used for seven years in early RA did not significantly change safety outcomes, indicating a good safety profile for very low-dose GCs [30]. In our sample, the majority of GC users perceived GCs as unsafe, and more than half of our sample used GCs for years. The above studies suggest that low-dose GCs may still be safe for patients, and therefore, patients should be educated about the risks and benefits of long-term GC use and be involved in their care decisions with their physicians.

The addition of low-dose GCs has also been shown to be cost-effective in some studies. Adding low-dose prednisolone was found to non-significantly reduce costs while significantly reducing disease activity [31]. Economic analysis of the Combination Anti-Rheumatic Drugs in Early RA (CARDERA) trial showed that the addition of short-term GCs, in addition to two DMARDs, resulted in decreased hospital costs over the course of two years when compared to DMARDs alone or one DMARD with GCs [32]. On the other hand, long-term GC usage in RMDs like antineutrophil cytoplasmic antibody (ANCA)-associated vasculitis was not found to lower relapse rates or improve survival. Still, it increases the risk of severe infections, especially with a daily dose above 7.5 mg [33].

These economic findings should be considered when considering treatment plans, particularly in developing countries like Jordan. Patients in developing countries may turn to therapies such as complementary medicine to lower their RMD therapy costs or for fear of side effects [34]. For instance, Jordanians were found to have significantly higher GC use and substantially lower use of TNF inhibitors and methotrexate as compared to neighboring countries, the majority of which have higher GDPs [12]. Although DMARDs are available in Jordan, and the Jordanian Food and Drug Administration is approving biosimilars [35], the costs of these more effective drugs are still high, and their availability is limited, particularly for the uninsured or those with private insurance rather than public insurance. Data from the Moroccan arthritis registry showed that the median annual cost for biologic therapy was 1665 euros per patient, and total direct annual costs were nearly one million euros, which indicates a degree of financial burden for developing countries [36]. Further studies are required to assess the cost-effectiveness of GC therapy in a developing country like Jordan, where biological treatment may have a considerable economic burden.

This study’s strengths include its large sample size and standardized recruitment by a single researcher at a tertiary referral center. However, its cross-sectional design limits the ability to establish causal relationships between glucocorticoid (GC) use and the variables being studied. The use of convenience sampling may introduce selection bias and limit generalizability, though resource constraints prevented a more randomized approach. Additionally, recall bias could affect data accuracy, as participants self-reported past GC use, adverse effects, and medication adherence without objective verification. Furthermore, recruitment was limited to the rheumatology department, excluding other specialties where the use of GCs is common. Future studies should aim for a broader patient population across multiple specialties to enhance the understanding of GC use and perceptions in Jordan.

## Conclusions

GC use is prevalent among patients with RMDs in Jordan, with many patients showing a strong awareness of the associated side effects. Perceived efficacy and safety of GCs are significantly influenced by education level and monthly income, suggesting that patients with higher socioeconomic status may be less likely to trust unreliable information sources. However, dependence on less credible sources may contribute to nonadherence and mistrust in GC safety. To improve patient safety, treatment adherence, and disease outcomes, healthcare professionals in Jordan should prioritize educating patients about GC side effects and safety profiles.

## References

[1] Buttgereit F. Views on glucocorticoid therapy in rheumatology: the age of convergence. Nat Rev Rheumatol. 2020;16(4):239–46. doi: 10.1038/s41584-020-0370-z 32076129

[2] Rice JB, White AG, Scarpati LM, Wan G, Nelson WW. Long-term Systemic Corticosteroid Exposure: A Systematic Literature Review. Clin Ther. 2017;39(11):2216–29. doi: 10.1016/j.clinthera.2017.09.011 29055500

[3] Seo MR, Kim G, Moon KW, Sung YK, Yoo JJ, Yoon CH, et al. Quality Indicators for Evaluating the Health Care of Patients with Rheumatoid Arthritis: a Korean Expert Consensus. J Korean Med Sci. 2021;36(17):e109. doi: 10.3346/jkms.2021.36.e109 33942576 PMC8093604

[4] Alnaimat F, Alduradi H, Al-Qasem S, Ghazzal H, Alsarhan M. Giant cell arteritis: insights from a monocentric retrospective cohort study. Rheumatol Int. 2024;44(6):1013–23. doi: 10.1007/s00296-024-05540-5 38502233

[5] Adwan MH, Qasem U, Atawnah SY, Itmeizeh M, Hanbali R, Alsoofi NA, et al. Insights into systemic lupus erythematosus: a retrospective observational study of clinical features, autoantibodies, and gender-related differences. Rheumatol Int. 2024;44(7):1255–63. doi: 10.1007/s00296-024-05592-7 38717538

[6] Hua C, Buttgereit F, Combe B. Glucocorticoids in rheumatoid arthritis: current status and future studies. RMD Open. 2020;6(1):e000536. doi: 10.1136/rmdopen-2017-000536 31958273 PMC7046968

[7] Nam B, Sung Y-K, Choi C-B, Kim T-H, Jun J-B, Bae S-C, et al. Fracture Risk and Its Prevention Patterns in Korean Patients with Polymyalgia Rheumatica: a Retrospective Cohort Study. J Korean Med Sci. 2021;36(41):e263. doi: 10.3346/jkms.2021.36.e263 34697929 PMC8546306

[8] Kim SY, Yoo C-G, Lee CT, Chung HS, Kim YW, Han SK, et al. Incidence and risk factors of steroid-induced diabetes in patients with respiratory disease. J Korean Med Sci. 2011;26(2):264–7. doi: 10.3346/jkms.2011.26.2.264 21286019 PMC3031012

[9] Smolen JS, Landewé RBM, Bergstra SA, Kerschbaumer A, Sepriano A, Aletaha D, et al. EULAR recommendations for the management of rheumatoid arthritis with synthetic and biological disease-modifying antirheumatic drugs: 2022 update. Ann Rheum Dis. 2023;82(1):3–18. doi: 10.1136/ard-2022-223356 36357155

[10] Fraenkel L, Bathon JM, England BR, St Clair EW, Arayssi T, Carandang K, et al. 2021 American College of Rheumatology Guideline for the Treatment of Rheumatoid Arthritis. Arthritis Care Res (Hoboken). 2021;73(7):924–39. doi: 10.1002/acr.24596 34101387 PMC9273041

[11] Sagtaganov Z, Yessirkepov M, Bekaryssova D, Suigenbayev D. Managing rheumatoid arthritis and cardiovascular disease: the role of physical medicine and rehabilitation. Rheumatol Int. 2024;44(9):1749–56. doi: 10.1007/s00296-024-05651-z 38914772

[12] Dargham SR, Zahirovic S, Hammoudeh M, Al Emadi S, Masri BK, Halabi H, et al. Epidemiology and treatment patterns of rheumatoid arthritis in a large cohort of Arab patients. PLoS One. 2018;13(12):e0208240. doi: 10.1371/journal.pone.0208240 30566451 PMC6300286

[13] Bergstra SA, Sepriano A, Kerschbaumer A, van der Heijde D, Caporali R, Edwards CJ, et al. Efficacy, duration of use and safety of glucocorticoids: a systematic literature review informing the 2022 update of the EULAR recommendations for the management of rheumatoid arthritis. Ann Rheum Dis. 2023;82(1):81–94. doi: 10.1136/ard-2022-223358 36410794

[14] Nassar K, Janani S, Roux C, Rachidi W, Etaouil N, Mkinsi O. Long-term systemic glucocorticoid therapy: patients’ representations, prescribers’ perceptions, and treatment adherence. Joint Bone Spine. 2014;81(1):64–8. doi: 10.1016/j.jbspin.2013.07.001 23953225

[15] مستشفى الجامعة الأردنية:: عمان:: الأردن:: Jordan University Hospital. [cited 19 Jun 2025]. Available: https://hospital.ju.edu.jo/AnnualReports/Forms/All_Reports.aspx

[16] Zimba O, Gasparyan AY. Designing, Conducting, and Reporting Survey Studies: A Primer for Researchers. J Korean Med Sci. 2023;38(48):e403. doi: 10.3346/jkms.2023.38.e403 38084027 PMC10713437

[17] von Elm E, Altman DG, Egger M, Pocock SJ, Gøtzsche PC, Vandenbroucke JP, et al. The Strengthening the Reporting of Observational Studies in Epidemiology (STROBE) statement: guidelines for reporting observational studies. J Clin Epidemiol. 2008;61(4):344–9. doi: 10.1016/j.jclinepi.2007.11.008 18313558

[18] Education Statistics | International Standard Classification of Education (ISCED). [cited 19 Jun 2025]. Available: https://datatopics.worldbank.org/education/wRsc/classification

[19] Mahdy A, Hussain N, Khalidi D, Said A. Knowledge, attitude, and practice analysis of corticosteroid use among patients: A study based in the United Arab Emirates. Natl J Physiol Pharm Pharmacol. 2017:1. doi: 10.5455/njppp.2017.7.1234409022017

[20] Qutob RA, Alhusaini BA, Aljarba NK, Alzaid ON, Aljahili NA, Alzahrani KS, et al. Public Awareness Regarding Corticosteroid Use and Side Effects: A Cross-Sectional Study in Riyadh, Saudi Arabia. Healthcare (Basel). 2023;11(20):2747. doi: 10.3390/healthcare11202747 37893821 PMC10606483

[21] Richards RN. Side effects of short-term oral corticosteroids. J Cutan Med Surg. 2008;12(2):77–81. doi: 10.2310/7750.2008.07029 18346404

[22] Costello R, Patel R, Humphreys J, McBeth J, Dixon WG. Patient perceptions of glucocorticoid side effects: a cross-sectional survey of users in an online health community. BMJ Open. 2017;7(4):e014603. doi: 10.1136/bmjopen-2016-014603 28373256 PMC5387953

[23] Cheah JTL, Robson JC, Black RJ, Goodman SM, Lester S, Mackie SL, et al. The patient’s perspective of the adverse effects of glucocorticoid use: A systematic review of quantitative and qualitative studies. From an OMERACT working group. Semin Arthritis Rheum. 2020;50(5):996–1005. doi: 10.1016/j.semarthrit.2020.06.019 32911291

[24] Deniz S, Akbolat M, Çimen M, Ünal Ö. The Mediating Role of Shared Decision-Making in the Effect of the Patient-Physician Relationship on Compliance With Treatment. J Patient Exp. 2021;8:23743735211018066. doi: 10.1177/23743735211018066 34179444 PMC8205395

[25] Abdelrahman W, Al-Shaarawy A, El-Zorkany B. Influence of perception of glucocorticoids on compliance of treatment in patients with rheumatoid arthritis and systemic lupus erythematosus. The Egyptian Rheumatologist. 2023;45(2):159–64. doi: 10.1016/j.ejr.2023.01.002

[26] Arena C, Morin A-S, Blanchon T, Hanslik T, Cabane J, Dupuy A, et al. Impact of glucocorticoid-induced adverse events on adherence in patients receiving long-term systemic glucocorticoid therapy. Br J Dermatol. 2010;163(4):832–7. doi: 10.1111/j.1365-2133.2010.09877.x 20518780

[27] Smith SD, Farrugia LL, Harris V, Lee A, Carter SR, Blaszczynski A, et al. Evaluation of the influence of family and friends, and the Internet on patient perceptions of long-term topical corticosteroid use. J Dermatolog Treat. 2017;28(7):642–6. doi: 10.1080/09546634.2017.1306017 28349719

[28] Stone JH, McDowell PJ, Jayne DRW, Merkel PA, Robson J, Patel NJ, et al. The glucocorticoid toxicity index: Measuring change in glucocorticoid toxicity over time. Semin Arthritis Rheum. 2022;55:152010. doi: 10.1016/j.semarthrit.2022.152010 35486995

[29] Boers M, Hartman L, Opris-Belinski D, Bos R, Kok MR, Da Silva JA, et al. Low dose, add-on prednisolone in patients with rheumatoid arthritis aged 65+: the pragmatic randomised, double-blind placebo-controlled GLORIA trial. Ann Rheum Dis. 2022;81(7):925–36. doi: 10.1136/annrheumdis-2021-221957 35641125 PMC9209692

[30] Roubille C, Rincheval N, Dougados M, Flipo R-M, Daurès J-P, Combe B. Seven-year tolerability profile of glucocorticoids use in early rheumatoid arthritis: data from the ESPOIR cohort. Ann Rheum Dis. 2017;76(11):1797–802. doi: 10.1136/annrheumdis-2016-210135 28213564

[31] Hartman L, El Alili M, Cutolo M, Opris D, Da Silva J, Szekanecz Z, et al. Cost-effectiveness and cost-utility of add-on, low-dose prednisolone in patients with rheumatoid arthritis aged 65+: The pragmatic, multicenter, placebo-controlled GLORIA trial. Semin Arthritis Rheum. 2022;57:152109. doi: 10.1016/j.semarthrit.2022.152109 36335684

[32] Wailoo A, Hernández Alava M, Scott IC, Ibrahim F, Scott DL. Cost-effectiveness of treatment strategies using combination disease-modifying anti-rheumatic drugs and glucocorticoids in early rheumatoid arthritis. Rheumatology (Oxford). 2014;53(10):1773–7. doi: 10.1093/rheumatology/keu039 24771112

[33] Speer C, Altenmüller-Walther C, Splitthoff J, Nusshag C, Kälble F, Reichel P, et al. Glucocorticoid maintenance therapy and severe infectious complications in ANCA-associated vasculitis: a retrospective analysis. Rheumatol Int. 2021;41(2):431–8. doi: 10.1007/s00296-020-04752-9 33222006 PMC7835159

[34] Alnaimat F, Alduraidi H, Alhafez L, Abu Raddad L, Haddad BI, Hamdan M, et al. Rates, patterns, and predictors of complementary medicine use among patients with musculoskeletal diseases. PLoS One. 2023;18(6):e0287337. doi: 10.1371/journal.pone.0287337 37352251 PMC10289458

[35] Alawneh K, Al-Mistarehi A-H, Qandeel A, Jaber R, Alomari S, Kheirallah KA. The Safety and Effectiveness of Infliximab Biosimilar in Managing Rheumatoid Arthritis: A Real-Life Experience from Jordan. Int J Clin Pract. 2022;2022:3406783. doi: 10.1155/2022/3406783 36101813 PMC9439897

[36] Fellous S, Rkain H, Ahid S, Abouqal R, Tahiri L, Hmamouchi I, et al. One-year direct costs of biological therapy in rheumatoid arthritis and its predictive factors: data from the Moroccan RBSMR registry. Rheumatol Int. 2021;41(4):787–93. doi: 10.1007/s00296-020-04762-7 33386900

